# Cigarette Prices and Smoking Behavior in Israel: Findings from a National Study of Adults (2002–2017)

**DOI:** 10.3390/ijerph18168367

**Published:** 2021-08-07

**Authors:** Kerem Shuval, Michal Stoklosa, Nigar Nargis, Jeffrey Drope, Shay Tzafrir, Lital Keinan-Boker, Laura F. DeFina, Mahmoud Qadan

**Affiliations:** 1 School of Business Administration, Faculty of Social Sciences, University of Haifa, Haifa 3498838, Israel; stzafrir@univ.haifa.ac.il (S.T.); mqadan@univ.haifa.ac.il (M.Q.); 2 School of Public Health, Faculty of Social Welfare & Health Sciences, University of Haifa, Haifa 3498838, Israel; lital.keinan2@moh.gov.il; 3 The Cooper Institute, Dallas, TX 75230, USA; ldefina@cooperinst.org; 4 Institute for Health Research and Policy, University of Illinois at Chicago, Chicago, IL 60608, USA; mjstoklosa@gmail.com; 5 Economic and Health Policy Research, American Cancer Society, Atlanta, GA 30303, USA; nigar.nargis@cancer.org; 6 Health Policy & Administration, School of Public Health, University of Illinois at Chicago, Chicago, IL 60608, USA; jdrope@uic.edu; 7 Israel Center for Disease Control, Ministry of Health, Sheba Medical Center, Ramat Gan 5262160, Israel

**Keywords:** cigarette prices, smoking, adults, Israel

## Abstract

Tobacco taxation and price policies are considered the most effective for lowering demand for tobacco products. While this statement is based on research from numerous countries, scant evidence exists on this topic for Israel. Accordingly, we assessed the association between cigarette prices and smoking prevalence and intensity from a national sample of adults in Israel (2002–2017). Data on smoking behavior were derived from the Israeli Knowledge Attitudes and Practices (KAP) survey, a repeated cross-sectional survey. Price information is from the Economist Intelligence Unit (EIU) since it was not collected in the KAP survey. We used the price of a pack of 20 cigarettes for Marlboro and the local brand. These two price variables were the primary independent variables, and we adjusted for inflation. The dependent variables were current smoking (yes/no) and smoking intensity, defined as the number of cigarettes smoked per week. Multivariable analysis was employed using a two-part model while adjusting for covariates. The first step of the model utilized logistic regression with current smoking as the dependent variable. The second step examining smoking intensity as the dependent variable, used OLS regression. Price elasticity was estimated as well. Analysis revealed that a one-unit increase (Israeli currency) in the price of local brand of cigarettes was related to 2.0% (OR = 0.98; 95%CI 0.98, 0.99) lower odds of being a current smoker, adjusting for covariates including household income. Moreover, a one unit increase in the price of the local brand of cigarettes was related to consuming 1.49 (95% CI −1.97, −1.00) fewer weekly cigarettes, controlling for household income and covariates. Similar results were found with the Marlboro cigarette prices. The total price elasticity of cigarette demand, given by the sum of price elasticities of smoking prevalence and intensity, showed that a 10.0% increase in the price is associated with a 4.6–9.2% lower cigarette consumption among Israeli adults. Thus, increasing cigarette prices will likely lead to a reduction in cigarette smoking thereby improving public health in Israel.

## 1. Introduction

Tobacco use is a leading cause of premature death worldwide, and the largest preventable risk factor of most major non-communicable diseases [1]. For example, tobacco use is the largest preventable cause for cancer morbidity and mortality. According to the National Cancer Institute, tobacco use is causally linked to at least 12 types of cancer, including lung, larynx, throat, pancreas, breast and colon [2]. Despite this fact, according to the World Health Organization (WHO) in 2015, 1.1 billion individuals smoked tobacco worldwide [3]. In Israel, based on the WHO Global Report on Trends in the Prevalence of Tobacco [4], the smoking prevalence decreased from 31.7% in 2000 to 29.0% in 2005, and then to 26.9% and 25.0% in 2010 and 2015, respectively.

The WHO predicts that smoking prevalence in Israel will further decrease to 21.9% by 2025, which reflects an 18.6% relative reduction from the level in 2010; thereby not meeting the 30.0% benchmark suggested by the WHO [4]. Furthermore, while national data from Israel indicate an overall declining prevalence of smoking (22.5% in 2016), it is still high, particularly compared to many other high-income countries [5]. Numerous countries have increased taxes to raise the prices of tobacco products to reduce the prevalence of tobacco use [6,7]. Research on low, middle, and high income countries has found that increasing cigarette prices is a cost-effective measure to lower smoking prevalence, thereby lowering the detrimental health and economic effects of smoking [8,9]. Currently in Israel, the taxes on cigarettes are ~80.1% retail price compared to 80.8%, on average, in the European Union (EU) [10]. Though this is above the WHO recommendation of 75% [11], a more relevant measure is the absolute price [8]. If the prices remain low regardless of the tax share, they are going to affect consumption positively. Of note, in terms of tax structure, is the change in the tax base for ad valorem from retail price to wholesale price in 2009.

Indeed, the WHO Framework Convention on Tobacco Control (WHO FCTC) states that taxation and price policies are the most effective strategy in reducing demand for tobacco and should be implemented alongside other tobacco control strategies (e.g., restrictions of smoking in public places, warning labels on packages, and marketing restrictions) [10,12]. Despite the importance of tobacco taxation and prices on individuals’ tobacco use behavior, to our knowledge, no scientific peer-reviewed research study exists in Israel, beyond an important ‘white paper’ on this topic [13]. Hence, in the current study we examine the relationship between cigarette price variability and smoking behavior in Israel. More specifically, we assess whether cigarette prices were related to the prevalence and intensity of smoking from 2002 to 2017 among adults in Israel.

## 2. Materials and Methods

### 2.1. Data and Design

This study utilizes a repeated cross-sectional design to explore the association between cigarette prices and smoking prevalence and intensity. Data on tobacco were derived from the Israeli Knowledge Attitudes and Practices (KAP) survey, which utilizes a stratified random sample design [14,15]. The KAP is a national repeated cross-sectional survey of the Israeli adult population conducted over the phone. The KAP, conducted by the Israeli Center for Disease Control since 2000, aims to describe health behaviors (e.g., smoking, physical activity) of adults in Israel every few years [14,16]. In the current study, we examined adults (≥18 years) who completed the KAP survey 2002–2017. There were 7 surveys during this period: 2002, 2004, 2006, 2008, 2011, 2013, and 2017. These surveys were pooled together in the current study to allow for greater variability in aggregate level cigarette prices over time and identify the relationship of price to cigarette smoking prevalence (see Measures). A total of 32,540 participants completed the survey and had complete information on the primary study variables except for household income. The analytic sample of models adjusting for income (see statistical analysis) was smaller (*n* = 24,492) since this variable was not queried in the KAP 2002 wave and due to missing responses or not disclosing income.

### 2.2. Measures

Smoking variables and Cigarette Prices. Current smoking (daily, sometimes, no) and smoking intensity were based on self-report from the KAP survey. The current smoking variable was dichotomized into a ‘yes/no’ binary variable, and smoking intensity was defined as the number of cigarettes smoked, on average, per week. In addition, price information was obtained from the Economist Intelligence Unit (EIU) and matched with KAP data by year of survey since it was not collected as part of the KAP survey [17]. Hence, the price of cigarettes for each of the 7 survey waves (2002–2017) was derived from the EIU, which collects cigarette prices worldwide over time [17]. Specifically, we examined the price, in Israeli currency (New Israeli Shekel—NIS), of a pack of 20 cigarettes for Marlboro and for the local brand. Subsequently, two price variables were regarded as the primary independent variables, both adjusted for inflation rates.

Covariates. These included participants’ sex, age (18–24, 25–64, ≥65 years), ethnicity (Jews/Arabs), religiosity (secular, traditional, religious, ultra-orthodox), being married (yes/no), and college education (yes/no). Additionally, analyses accounted for reported monthly household income: below average, about average, or above average in comparison to the average national income at the time of the KAP survey (see footnote in the first table).

### 2.3. Statistical Analysis

Descriptive statistics were used to characterize study variables. We employed a two-step model approach, which is consistent with other studies in the field [18], to estimate the relationship of cigarette prices (Marlboro and the local brand, separately) to smoking prevalence and intensity. In the first step of the model, logistic regression was used to examine the association between prices as well as covariates and current smoking (binary: yes/no). Odds ratios (OR) and 95% confidence intervals (CI) were computed for the logistic regression models. The second step of the model consisted of only current smokers and estimated the association of cigarette prices with the number of cigarettes consumed per week using ordinary least-squares (OLS) models; beta coefficients and 95% CI were computed for these models. A total of two multivariable models were constructed for both logistic and OLS regression. The first model included participants’ age, sex, ethnicity, religiosity, marital status, college education, and survey year as a dummy variable for each survey year with the exception of 2017, which was omitted from the model due to collinearity. The second model included covariates from the first model as well as household income. Additionally, when examining the interaction of cigarette price X income (below vs. average/above average) with smoking prevalence, the terms for the local brand and Marlboro were statistically significance (*p* < 0.01). Hence, separate models were constructed stratified by income level for the local brand and Marlboro, where smoking prevalence was the dependent variable. Finally, the ORs from the logistic regression modes and the coefficients from the OLS models were converted into price elasticities [9]. Price elasticity is a standard economic measure, which assesses how sensitive the demand for a given good or service is to changes in its price. The price elasticities were estimated using the “margins” function in the statistical program, STATA 1SE V.15.1 (Stata-Corp LP, College Station, TX, USA). The function estimates margins at the means of covariates ($\frac{\partial y}{\partial x}\times \frac{\bar{x}}{\bar{y}})$.

## 3. Results

Participants’ characteristics are described in Table 1 for the entire sample and by survey year. For the overall sample, slightly more than half (53.7%) were women, 17.6% of participants were 65 years or older, and 31.0% were college graduates. In addition, 47.7% reported an income that was about at the national average household income in Israel or higher than the national average. Of the participants, 63.8% were Jews, 36.2% were Arabs, while 77.0% reported being married. In terms of religiosity, 20.8% defined themselves as religious and 6.1% ultra-orthodox. More than a fifth (21.6%) were current smokers, and the average number of cigarettes smoked per week was 114.4 (SD = 84.6). Participants’ characteristics varied by survey wave and are presented in Table 1.

**Figure 1:**
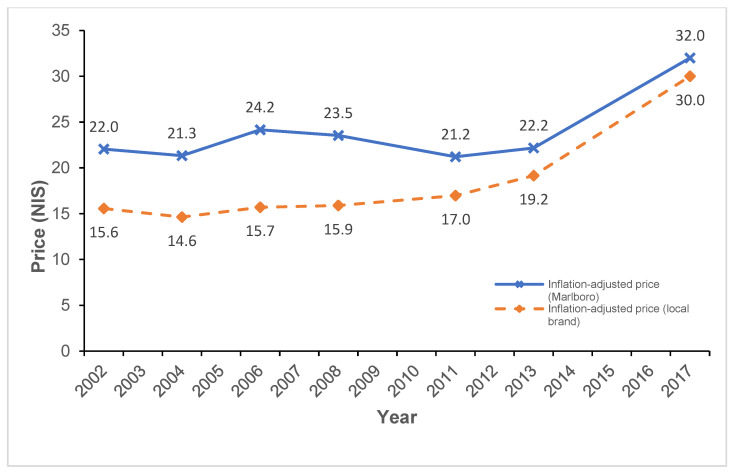
Cigarette Prices (Marlboro and Local Brand) by Survey Year. The blue ‘x’ and orange ‘rhombus’ shape depict the inflation adjusted price in NIS (New Israeli Shekel) of a 20 pack of cigarettes of Marlboro and the local brand (respectively) per KAP (knowledge, attitudes and practices) survey year: 2002, 2004, 2006, 2008, 2011, 2013, 2017. The base year for inflation adjustment is 2017. Prices were derived from the Economic Intelligence Unit.

In addition, inflation adjusted cigarette prices for the local brand and Marlboro (pack of 20), as derived from the EIU, by KAP survey year, appear in Figure 1. As depicted in the figure, in 2002 the inflation adjusted price of the local brand of cigarettes was 15.6 NIS, whereas the price of the local brand was 22.0 NIS. In comparison, in 2017, the price of the local brand was 30.0 NIS, and the price of Marlboro was 32.0 NIS.

The relationship between cigarette prices (local brand, Marlboro), covariates, and the prevalence of current smoking, examined via logistic regression models, is depicted in Table 2. The association between the price of the local cigarette brand and the prevalence of current smoking was statistically significant in all models (*p* < 0.01 in all models). Specifically, a one unit increase in Israeli currency (1 NIS = ~0.31 US dollar in 2021) of the local brand of cigarettes was related to a 1.0% (OR= 0.99; 95% CI 0.98, 1.00), and 2.0% (OR= 0.98; 95% CI 0.98, 0.99) lower odds of being a current smoker, when adjusting for covariates, and covariate plus income, respectively. Similar results were found when examining the relation between the price of Marlboro cigarettes and the prevalence of smoking in the first model (OR = 0.99; 95% CI 0.98, 1.00), and the second model which controlled for income (OR = 0.98; 95% CI 0.97, 0.99). The multivariable relationship between covariates and smoking prevalence is also depicted in Table 2. For example, in the second model, women had lower odds for current smoking than men (OR = 0.35; 95% CI 0.32, 0.37), whereas Arab participants had higher odds for current smoking than Jewish participants (OR = 1.13; 95% CI 1.05, 1.22). Additionally, participants with about average and above average income had significantly higher odds for current smoking than their below average income counterparts (about average: OR = 0.87; 95% CI 0.80, 0.95; above average: OR = 0.76; 95% CI 0.69, 0.84); this is while holding cigarette prices and other covariates constant (i.e., in multivariable analysis).

When stratifying the analysis by income (adjusting for covariates), results show significantly lower odds of current smoking with increased cigarette prices among participants with about average and above average income levels (Table 3). Specifically, a 1-NIS increase in the price of local brand of cigarettes was related with 2.0% (OR= 0.98; 95%CI 0.97, 1.00) lower odds for current smoking among participants with about average incomes, as well as 2.0% (OR = 0.98; 95%CI 0.97, 0.99) lower odds for those with above average income levels. Similarly, a 1-NIS increase in the price of Marlboro cigarettes was associated with 2.0% (OR = 0.98; 95%CI 0.96, 1.00) lower odds of current smoking for participants with about average incomes, and 3.0% (OR = 0.97; 95%CI 0.95, 0.99) lower odds for participants with above average incomes. When examining participants with below average incomes, these relationships were not statistically significant (see Table 3).

The multivariable association between cigarette prices and smoking intensity among current smokers using OLS regression is presented in Table 4. Analysis revealed that a 1-NIS increase in the price of the local cigarette brand was related to consuming 1.92 (95% CI −2.39, −1.45) fewer cigarettes per week, and 1.49 (95%CI −1.97, −1.00) fewer weekly cigarettes when adjusting for covariates and covariates plus income, respectively. Similar findings were observed when assessing the association between the price for Marlboro cigarettes and smoking intensity. Specifically, a 1-NIS increase in the price of Marlboro cigarettes was associated with 2.79 (95%CI −3.47, −2.10) and 2.14 (95%CI −2.84, −1.45) fewer weekly cigarettes adjusting for covariates and covariates and income, respectively. The relationship between covariates and smoking intensity is also presented in Table 4. For example, Arab participants smoked 23.53 (95%CI 18.97, 28.09) and 22.08 (95%CI 16.91, 27.24) more cigarettes per week than Jewish participants adjusting for covariates and covariates and income, respectively. Moreover, college graduates smoked 32.05 (95%CI −36.69, −27.42) and 29.71 (95%CI −35.10, −24.32) fewer cigarettes per week than non-graduates adjusting for covariates and covariates and income, respectively.

In addition, the estimated price elasticities of smoking prevalence, which correspond to the ORs in the logistic regression, vary from −0.15 to −0.47. The estimated price elasticity of smoking intensity, which correspond to coefficients from the OLS regression, vary from −0.25 to −0.58. The total price elasticity of cigarette demand in Israel, obtained by adding elasticity of smoking prevalence and elasticity of smoking intensity in the corresponding models, vary from −0.46 to −0.92. This means that a 10.0% increase in the price of cigarettes is expected to lead to a 4.6% to 9.2% lower cigarette consumption among Israeli adults.

## 4. Discussion

To our knowledge, this is the first study in Israel to examine the relationship between individual level smoking behavior in adults and cigarette prices. Specifically, the present study explores whether increases in cigarette prices are related to lower odds for smoking, and lower levels of smoking intensity among current smokers. Our analysis, which employed a two-part model, demonstrates that higher cigarette prices were related to lower odds of being a current smoker among Israeli adults, and that higher cigarette prices were associated with lower smoking intensity. Furthermore, when estimating price elasticities, we observed that a 10.0% increase in the price of cigarettes is related to a 4.6% to 9.2% reduction in cigarette consumption in Israel. This is consistent with other high-income countries where most estimates of elasticities of demand range from 2.0–6.0%, clustering around 4.0% [9]. This finding has implications for policy makers regarding the size of a price increase needed to reduce smoking which is paramount to lowering morbidity and mortality risk [19].

Moreover, present results showing that higher cigarette prices are associated with lower odds of current smoking is consistent with a large body of scientific research [9]. Specifically, a 1-NIS increase in cigarette prices was linked to 2.0% lower odds of being a current smoker (adjusting for income and covariates). This finding is similar to that found in a study by Kalousova et al. in the US, where a 1 dollar increase in local price was associated with 0.6% decrease in current smoking [20]. Levy et al. emphasize that large increases in cigarette taxes is the most robust policy lever to reduce smoking alongside other strategies, such as smoke free laws, marketing bans, and media campaigns [21]. Hence, increasing excise taxes on cigarettes in Israel even further, leading to increase cigarette prices, will most likely reduce the prevalence of smoking beyond its current levels.

The Cigarette Tax Scorecard, which scores cigarette tax policy performance in more than 170 countries (on a five-point scale), provided a relatively good score for Israel [8]. The score for Israel was 3.63 in 2018, which was above the world average (2.07) and above the average for high-income countries (2.85), but still well below the score for best performing countries (Australia and New Zealand, each having a score of 4.63) [8]. The Scorecard identifies areas of improvement for Israel. Specifically, although the 2009 tax change increased the tax rate for the specific portion of the excise tax while decreasing the ad valorem tax rate, the system still mostly relies on the ad valorem tax. This allows for large variations in prices on the market [13], which undermines the effectiveness of the tax as a consumption reduction measure. Furthermore, the switch from retail to whole price as the base for the ad valorem tax may also be giving the tobacco industry opportunities to adjust wholesale prices that undermine tax policy. Moreover, increases in Israelis’ incomes might partially offset the effects of tax/price increases on cigarette consumption, hence adjusting for economic growth should be considered. Future tobacco tax increases should raise cigarette prices significantly, so that the cigarette affordability is substantially reduced.

Importantly, current findings show that household income should be considered when examining the relationship between cigarette prices and smoking prevalence. When stratifying analysis by income level, the strength of the relationship differed, indicating that income appears to be an effect modifier, which is consistent with the significant interaction terms observed for the local cigarette brand and Marlboro. Specifically, our results indicate that higher cigarette prices are markedly related to lower odds of current smoking in individuals of moderate and high-income, but not significantly in participants of low-income. This finding contradicts prior studies reporting that individuals of low-income exhibit greater price sensitivity than their higher income counterparts [20,22]. A study by Sharbaugh et al., however, similarly observed that cigarettes taxes had the least impact on the prevalence of smoking among low-income US adults [23], suggesting that this population might engage in price minimization behaviors, such as the use of roll-your-own cigarettes [24]. This explanation is pertinent to the present study since the retail price of factory-made cigarettes has been estimated to be twice as high as roll-your-own cigarettes, due to significantly higher taxes for factory made cigarettes, as of 2017 [13].

A recent (2019) major regulatory change occurred in Israel (as a result of legislation a year prior), in which taxation on factory-made cigarettes and roll-your-own were equalized [25]; this occurred after the study period (2002–2017). Please note that during the study period a number of tobacco control policy changes took place, which have been described in detail elsewhere [10]. Briefly, there was an expansion of marketing restrictions and the prohibition of selling tobacco to minors in 2004; expansion of smoke free legislation to include bars as well as improvement in enforcement in 2007; coverage of smoking cessation services by Israel’s universal health care (‘national basket of services’) in 2010; and smoke-free legislation expanded in 2012 [10]. Alongside increasing cigarette taxes, comprehensive tobacco control policy measures are instrumental in driving down smoking prevalence [21]. Accordingly, we attempted to add the non-tax elements of the WHO’s MPOWER package (i.e., MPOWE) into the analysis [26]. MPOWER comprises of best policy level practices aimed at lowering tobacco demand [27]. We believe that the lack of variation in the MPOWE scores was the main reason that we could not generate significant findings for these additional policy-level control variables. Thus, in the final presentation we exclude these policy variables. The effects of these and other social factors (e.g., peer effects) should be further investigated in future research but will almost certainly require more variation than was present for this study.

The current study has limitations stemming from the use of existing data. For example, individual level purchasing prices of cigarettes was not available, which necessitated using the aggregate EIU price data instead. The variability of this price measure is limited to national level changes over time. As a result, the effect of cross-sectional variation in cigarette prices is not reflected in the estimated price elasticities. Furthermore, the KAP survey did not comprehensively collect information on roll-your-own cigarette use or the use of other tobacco products. Additionally, the income variable utilized, differed in some survey waves, and was missing altogether in 2002. Moreover, since income was missing or not disclosed by some participants, the analytic samples taking income into account were smaller than the overall sample. Further, the KAP study did not provide survey weights which were therefore not included in the analyses; this could impact the national representativeness of estimates. Finally, the current study utilized cross-sectional data rather than longitudinal information, which was not available. Longitudinal data is necessary to estimate long-term price elasticity, which is not possible in the current study.

## 5. Conclusions

In summary, study results emphasize the role cigarette prices play in the smoking behavior of adults in Israel. Specifically, our analyses underscore the link between higher cigarette prices and a lower prevalence of current smoking, as well as lower intensity levels among smokers in Israel. Overall, raising cigarette prices by 10.0% is related to reduced cigarette consumption by 4.6% to 9.2%. With the price of the local cigarette brand (pack of 20) at 30 NIS in 2021 (source: EIU), a mere 1-NIS increase in price would correspond with a 1.5% to 3.1% reduction in cigarette consumption. These findings provide evidence to Israeli policy makers pertaining to the importance of raising cigarette taxes to increase prices and subsequently lower consumption with the ultimate goal of reducing the burden of non-communicable diseases.

## Figures and Tables

**Table 1:** Characteristics of study sample: entire sample and by survey wave (KAP 2002–2017).

VARIABLES	Entire Sample (*n* = 32,540) Percent	KAP2002 (*n* = 4358) Percent ^a^	KAP2004 (*n* = 4306) Percent ^a^	KAP2006 (*n* = 3113) Percent ^a^	KAP2008 (*n* = 4118) Percent ^a^	KAP2011 (*n* = 4765) Percent ^a^	KAP2013 (*n* = 5881) Percent ^a^	KAP2017 (*n* = 5999) Percent ^a^
**Age (years)**								
18–24	7.1%	11.6%	8.6%	9.3%	5.0%	5.0%	4.8%	6.3%
25–64	75.4%	72.2%	75.8%	72.7%	77.2%	77.6%	76.1%	74.9%
≥65	17.6%	16.2%	15.7%	18.0%	17.8%	16.7%	19.0%	18.9%
**Sex**								
Women	53.7%	57.1%	55.4%	57.5%	52.8%	52.4%	50.2%	53.0%
Men	46.3%	42.9%	44.6%	42.5%	47.2%	47.6%	49.8%	47.0%
**Ethnicity**								
Arab	36.2%	21.2%	22.9%	26.0%	38.1%	44.6%	47.7%	42.8%
Jew	63.8%	78.8%	77.1%	74.0%	61.9%	55.4%	52.3%	57.2%
**Household Income ^b^**								
Above average	26.4%	N/A	51.4%	26.8%	25.7%	26.8%	12.2%	22.4%
About average	21.3%	N/A	30.9%	23.9%	26.7%	20.0%	14.2%	17.1%
Below average	52.2%	N/A	17.7%	49.3%	47.6%	53.2%	73.6%	60.4%
**Marial Status**								
Married	77.0%	69.7%	72.6%	71.1%	80.1%	82.1%	83.1%	76.2%
Not married	23.0%	30.3%	27.4%	28.9%	19.9%	17.9%	16.9%	23.8%
**College Graduate**								
Yes	31.0%	24.1%	30.1%	32.2%	31.1%	29.3%	30.7%	37.7%
No	69.0%	75.9%	69.9%	67.8%	68.9%	70.7%	69.3%	62.3%
**Religiosity**								
Secular	39.3%	48.3%	45.7%	46.3%	39.1%	32.0%	31.7%	37.9%
Traditional	33.8%	32.3%	32.3%	32.2%	34.6%	35.2%	34.6%	34.5%
Religious	20.8%	13.2%	16.8%	16.5%	21.2%	26.1%	25.9%	22.0%
Ultra-orthodox	6.1%	6.2%	5.2%	5.0%	5.0%	6.7%	7.8%	5.7%
**Current Smoking**								
Yes	21.6%	23.9%	24.6%	21.3%	21.9%	21.4%	19.4%	20.3%
No	78.4%	76.1%	75.4%	78.7%	78.1%	78.6%	80.6%	79.7%
**Num. of Cigarettes per Week:** mean (SD)	114.4 (84.6)	124.4 (88.0)	114.4 (84.0)	109.2 (83.5)	114.0 (82.7)	118.4 (83.4)	118.1 (84.9)	102.2 (83.6)

Abbreviations: Num., number, NIS, New Israeli Shekel; KAP, Knowledge, Attitudes and Practices Survey; SD, standard deviation; N/A, not available. Notes: ^a^ When percentages are not 100% that is due to rounding; ^b^ Income sample size (for the entire sample): 24,492. Wave 2002—income not asked. Wave 2004–2011—based on participants’ responses to whether household income was above, below, or about the national average; participants were provided with the average household income at the time of the survey. Waves 2013 and 2017—participants were provided with numeric values and asked to select their house income. This selection was coded by investigators as below average, about average, and above average in comparison to the mean household income in Israel during the survey wave as derived from the Israeli Central Burau of Statistics.

**Table 2:** Cigarette Prices and Smoking Prevalence among Adults in Israel ^a^: Logistic Regression ^b^.

Independent Variables	Current Smoking	
	Model 1 OR (95%CI)	Model 2 OR (95%CI)
**Marlboro**—Cigarette Price ^c^	0.99 (0.98, 1.00) **	0.98 (0.97, 0.99) **
**Local Brand**—Cigarette Price ^c^	0.99 (0.98, 1.00) **	0.98 (0.98, 0.99) **
**Age** (ref. 18–24 years)		
25–64 years	1.73 (1.54, 1.95) **	1.54 (1.33, 1.78) **
≥65 years	0.64 (0.56, 0.74) **	0.58 (0.49, 0.69) **
**Sex** (ref. men)		
Women	0.37 (0.35, 0.39) **	0.35 (0.32, 0.37) **
**Ethnicity** (ref. Jew)		
Arab	1.20 (1.13, 1.28) **	1.13 (1.05, 1.22) **
**Household income** (ref. below average)		
About average	N/A	0.87 (0.80, 0.95) **
Above average	N/A	0.76 (0.69, 0.84) **
**Marital status** (ref. not married)		
Married	0.61 (0.57, 0.66) **	0.64 (0.59, 0.70) **
**College education** (ref. non-graduate)		
College graduate	0.52 (0.49, 0.56) **	0.56 (0.51, 0.60) **
**Religiosity** (ref. secular)		
Traditional	0.83 (0.78, 0.89) **	0.82 (0.76, 0.89) **
Religious	0.48 (0.44, 0.53) **	0.48 (0.43, 0.53) **
Ultra-orthodox	0.30 (0.25, 0.35) **	0.28 (0.23, 0.34) **

** *p* < 0.01. Abbreviations: Ref., reference group; OR, odds ratio; CI, confidence interval; N/A, not applicable. Notes: ^a^ KAP data 2002–2017. Sample size for Model 1: *n*= 32,540, and Model 2: *n* = 24,492, which additionally includes household income. ^b^ Logistic regression models adjust for survey year (dummy variables) except for 2017, which was dropped from the model due to collinearity. Separate models were constructed for Marlboro and the local brand. ^c^ Marlboro and the local brand are indicative of the prices (2002–2017) of 20 pack cigarettes (adjusted for inflation) in NIS as derived from the Economic Intelligence Unit.

**Table 3:** Cigarette Prices ^a^ and Smoking Prevalence Stratified by Household Income ^b^: KAP 2002–2017: Logistic Regression ^c^.

VARIABLES	OR	95%CI
**Marlboro**		
Below average income	0.98	0.96, 1.00
About average income	0.98 *	0.96, 1.00
Above average income	0.97 **	0.95, 0.99
**Local Brand**		
Below average income	0.99	0.97, 1.00
About average income	0.98 *	0.97, 1.00
Above average income	0.98 **	0.97, 0.99

* *p* < 0.05, ** *p* < 0.01. Abbreviations: KAP, Knowledge, Attitudes and Practices Survey; OR, odd ratio; CI, confidence interval. Notes: ^a^ inflation adjusted prices. Price data were derived from the Economic Intelligence Unit; ^b^ a total of 24,492 participants had household income information; ^c^ the logistic regression adjusts for sex, age, ethnicity, religiosity, marital status, college education, and survey year (dummy) except for 2017 which was omitted due to collinearity. P-values for interaction between price (Marlboro and local brand) and income (below vs. average/above average): *p* < 0.01 for both models.

**Table 4:** Cigarette Prices and Smoking Intensity among Adult Smokers in Israel ^a^: OLS regression ^b^.

Independent Variables	Smoking Intensity ^d^	
	Model 1 Coefficient (95%CI)	Model 2 Coefficient (95%CI)
**Marlboro**—Cigarette Price ^c^	−2.79 (−3.47, −2.10) **	−2.14 (−2.84, −1.45) **
**Local Brand**—Cigarette Price ^c^	−1.92 (−2.39, −1.45) **	−1.49 (−1.97, −1.00) **
**Age** (ref. 18–24 years)		
25–64 years	37.40 (29.50, 45.30) **	32.80 (23.06, 42.54) **
≥65 years	34.22 (24.64, 43.80) **	28.82 (17.25, 40.38) **
**Sex** (ref. men)		
Women	−32.21 (−36.44, −27.99) **	−35.36 (−40.24, −30.48) **
**Ethnicity** (ref. Jew)		
Arab	23.53 (18.97, 28.09) **	22.08 (16.91, 27.24) **
**Household income** (ref. below average)		
About average	N/A	−7.32 (−13.21, −1.43) *
Above average	N/A	−12.38 (−18.62, −6.14) **
**Marital status** (ref. not married)		
Married	−12.68 (−17.45, −7.91) **	−11.11 (−16.78, −5.45) **
**College education** (ref. non-graduate)		
College graduate	−32.05 (−36.69, −27.42) **	−29.71 (−35.10, −24.32) **
**Religiosity** (ref. secular)		
Traditional	−0.92 (−5.38, 3.53)	−0.11 (−5.21, 4.99)
Religious	−6.48 (−12.67, −0.28) *	−7.08 (−13.93, −0.23) *
Ultra-orthodox	−22.72 (−34.86, −27.42) **	−24.10 (−37.74, −10.47) **

* *p* < 0.05, ** *p* < 0.01. Abbreviations: Ref., reference group; OLS, ordinary least squares; CI, confidence interval; N/A, not applicable. Notes: ^a^ conditional upon a positive current smoking status (KAP 2002–2017). Sample size for Model 1: *n*= 6919, and Model 2: *n* = 5267, which additionally includes household income. ^b^ OLS regression models were estimated for smoking intensity. These models adjust for survey year (dummy variables) except for 2017, which was dropped from the model due to collinearity. Separate models were constructed for Marlboro and the local brand. ^c^ Marlboro and the local brand are indicative of the prices (2002–2017) of 20 pack cigarettes (adjusted for inflation) in NIS as derived from the Economic Intelligence Unit. ^d^ The number of cigarettes smoked, on average, per week.

## Data Availability

Survey data from the KAP (https://www.health.gov.il/English/MinistryUnits/ICDC/Health_Surveys/Pages/KAP.aspx- accessed on 6 August 2021) are available upon reasonable request (and pertinent review and approval) from the Israel Center for Disease Control, Ministry of Health (https://www.health.gov.il/English/MinistryUnits/ICDC/Pages/about.aspx- accessed on 6 August 2021) by email (icdc@moh.health.gov.il).
